# Prehospital on-scene anaesthetist treating severe traumatic brain injury patients is associated with lower mortality and better neurological outcome

**DOI:** 10.1186/s13049-019-0590-x

**Published:** 2019-01-28

**Authors:** Toni Pakkanen, Jouni Nurmi, Heini Huhtala, Tom Silfvast

**Affiliations:** 1 FinnHEMS Ltd, Research and Development Unit, Vantaa, Finland; 2 Faculty of Medicine and Life Sciences, University of Tampere, Tampere, Finland; 3 Emergency Medicine and Services, Helsinki University Hospital and Department of Emergency Medicine, University of Helsinki, Helsinki, Finland; 4 Faculty of Social Sciences, University of Tampere, Tampere, Finland; 5 Department of Anaesthesia and Intensive Care, Helsinki University Hospital, University of Helsinki, Helsinki, Finland

**Keywords:** Prehospital emergency care (MeSH), Emergency medical services (MeSH), Critical care (MeSH), Traumatic brain injury (MeSH), Airway management (MeSH), Endotracheal intubation (MeSH), Patient outcome assessment (MeSH), Glasgow outcome scale (MeSH)

## Abstract

**Background:**

Patients with isolated traumatic brain injury (TBI) are likely to benefit from effective prehospital care to prevent secondary brain injury. Only a few studies have focused on the impact of advanced interventions in TBI patients by prehospital physicians. The primary end-point of this study was to assess the possible effect of an on-scene anaesthetist on mortality of TBI patients. A secondary end-point was the neurological outcome of these patients.

**Methods:**

Patients with severe TBI (defined as a head injury resulting in a Glasgow Coma Score of ≤8) from 2005 to 2010 and 2012–2015 in two study locations were determined. Isolated TBI patients transported directly from the accident scene to the university hospital were included. A modified six-month Glasgow Outcome Score (GOS) was defined as death, unfavourable outcome (GOS 2–3) and favourable outcome (GOS 4–5) and used to assess the neurological outcomes. Binary logistic regression analysis was used to predict mortality and good neurological outcome. The following prognostic variables for TBI were available in the prehospital setting: age, on-scene GCS, hypoxia and hypotension. As per the hypothesis that treatment provided by an on-scene anaesthetist would be beneficial to TBI outcomes, physician was added as a potential predictive factor with regard to the prognosis.

**Results:**

The mortality data for 651 patients and neurological outcome data for 634 patients were available for primary and secondary analysis. In the primary analysis higher age (OR 1.06 CI 1.05–1.07), lower on-scene GCS (OR 0.85 CI 0.79–0.92) and the unavailability of an on-scene anaesthetist (OR 1.89 CI 1.20–2.94) were associated with higher mortality together with hypotension (OR 3.92 CI 1.08–14.23). In the secondary analysis lower age (OR 0.95 CI 0.94–0.96), a higher on-scene GCS (OR 1.21 CI 1.20–1.30) and the presence of an on-scene anaesthetist (OR 1.75 CI 1.09–2.80) were demonstrated to be associated with good patient outcomes while hypotension (OR 0.19 CI 0.04–0.82) was associated with poor outcome.

**Conclusion:**

Prehospital on-scene anaesthetist treating severe TBI patients is associated with lower mortality and better neurological outcome.

## Background

The incidence of patients admitted to hospital with traumatic brain injury (TBI) in Europe is estimated to be 262/100,000, with average related mortality of 11/100,000 [1]. Approximately 10–20% of all TBIs are moderate or severe, requiring intensive care unit treatment [2, 3]. Severe traumatic brain injury is defined as a head injury resulting in a Glasgow Coma Score of ≤8 [4] and the prognosis for severe TBI is that one in two patients dies as a result or is severely affected as a result of the trauma [5, 6]. In large registry studies, TBI outcomes have been demonstrated to be strongly associated with demographic and trauma-related factors (i.e., age, motor score, pupillary reactivity and computed tomography classification) as well as with secondary factors (hypoxia and arterial hypotension primarily) in large registry studies [6–8].

Prehospital assessment and treatment is an important link in providing appropriate care [9] as the prognosis of patients with severe TBI strongly depends on early support of vital functions [10, 11]. In particular, prehospital prevention of hypotension and hypoxia by adequate treatment including a secured airway, normoventilation and prevention of aspiration is strongly associated with improved outcome [12–15].

The effect of advanced interventions by prehospital physicians on patient outcomes has been examined in only a few controlled studies. Increased survival has been found in patients with major trauma and in cardiac arrest patients [16]. In particular, patients with isolated TBI are also likely to benefit from a prehospital physician treating and preventing secondary brain injury insults [17]. Severe TBI patients treated by on-scene anaesthetists have been shown to have a better prognosis in our previous studies [18, 19]. Thus, the current study objective was to further analyse the previously gathered patient data using binary logistic regression analysis. The hypothesis was that interventions by prehospital anaesthetists would have a positive effect on severe TBI patient outcomes. The primary end-point was to evaluate the possible effect of an on-scene anaesthetist on mortality and as a secondary end-point, the neurological outcome in TBI patients.

## Methods

### Study setting

The prehospital treatment and outcomes of patients with severe TBI from 2005 to 2010 and 2012–2015 in two study locations (Helsinki and Uusimaa region and Pirkanmaa region, Finland) were determined in this retrospective cohort study. The Helsinki and Uusimaa area represents a 10-year continuous patient flow in a physician-staffed emergency medical service (EMS) system. The Pirkanmaa patient cohort was divided into two sections: 2005–2010 with no prehospital physician service and 2011–2015 after the implementation of a physician-staffed EMS unit. Previously gathered patient data, in conjunction with previously unused data (representing 18% of the total information), was further analysed using binary logistic regression analysis. The data covering 2011 were excluded as a physician-staffed helicopter emergency medical service (HEMS) was implemented in the Pirkanmaa Hospital District that year and impacted significantly on the local EMS. There were no dedicated medical directors in the Pirkanmaa area until 2010 and EMS crews consulted on-call hospital physicians for treatment guidelines.

The two present EMS systems, described in detail in previous publications [18, 19], serve a total of almost two million inhabitants and comprise basic life support, advanced life support and physician-staffed units. The physician-staffed units respond to medical emergencies as well as trauma calls. The prehospital physicians are anaesthesiologists with extensive experience in prehospital emergency medicine. All severe TBI patients in these regions are admitted to the region’s single university hospital and receive immediate neurosurgical care according to the national guidelines [20].

The study protocol was approved by the Regional Ethics Committee of the Pirkanmaa Hospital District (No. R15158). Permission to conduct the study was obtained from the research directors of Tampere University Hospital and Helsinki University Hospital. The study was registered in ClinicalTrials.gov (Identifier NCT02659046) (originally on 15 January 2016 and then updated on 12 December 2017).

### Definitions and data collection

Severe TBI was defined as a GCS score ≤ 8, occurring either on scene, during transportation or verified by an on-call neurosurgeon on admission to hospital [21]. Advanced airway management was defined as securing the airway with endotracheal intubation, a supraglottic airway device (laryngeal mask) or surgical airway. Hypoxia was defined as a SpO_2_ of ≤90% and hypotension as a systolic blood pressure (SBP) of ≤90 mmHg. The definitions are consistent with the latest edition of the Brain Trauma Foundation’s guidelines for the prehospital management of TBI [4].

Included patients were identified from the hospital records based on ICD-10 discharge diagnoses for TBI (S06.2-S06.6 and S06.8). The inclusion criterion for the study was severe, isolated TBI in patients transported directly from the accident scene to the university hospital. Non-Finnish citizens were excluded from the study since follow-up data were not available to perform a neurological outcome evaluation. Patients with multiple injuries and requiring surgical intervention (other than neurosurgery) were also excluded, as were those who were transferred from other hospitals (i.e., inter-hospital transfers).

Age, gender, response time, total prehospital time, mechanism of injury, Glasgow Coma Scale (GCS) score, advanced airway management and vital signs on scene and on arrival at the emergency department (ED) were reviewed and cross-referenced with EMS run sheets and ED documentation.

Mortality data were obtained from the national statistical authority, Statistics Finland. A neurological outcome evaluation was performed based on the hospital patient records up to 6 months after the incident. A modified six-month Glasgow Outcome Score (GOS) [22, 23] was used to assess the neurological outcomes. A GOS of 1 denoted death within 6 months, a GOS of 2–3 was indicative of a poor neurological outcome (i.e., needing assistance with daily living activities) and a GOS of 4–5 was suggestive of good neurological recovery (i.e., the ability to lead an independent life). If the outcome was unclear, the research team members reviewed the case and a joint decision was made.

### Statistical methods

To describe general characteristics categorical variables are reported as percentage (%), while continuous variables are reported as median and range. Binary logistic regression analysis was used in univariate and multivariable models to predict mortality and a good neurological outcome. The evaluation was performed in the context of a prehospital environment using predictors that were of value in the prehospital treatment phase [17]. The following known conventional prognostic variables [5, 6] for TBI were available in the prehospital setting: age, on-scene GCS, hypoxia and hypotension. As per the hypothesis that treatment provided by an on-scene anaesthetist would be beneficial to TBI outcomes, physician was added as a potential predictive factor with regard to the prognosis. The results are presented as odds ratios (OR) with 95% confidence intervals. Statistical significance was considered to be a *p*-value of ≤0.050. The data were analysed using SPSS Statistics for Windows® version 21.0.

## Results

**Fig. 1:**
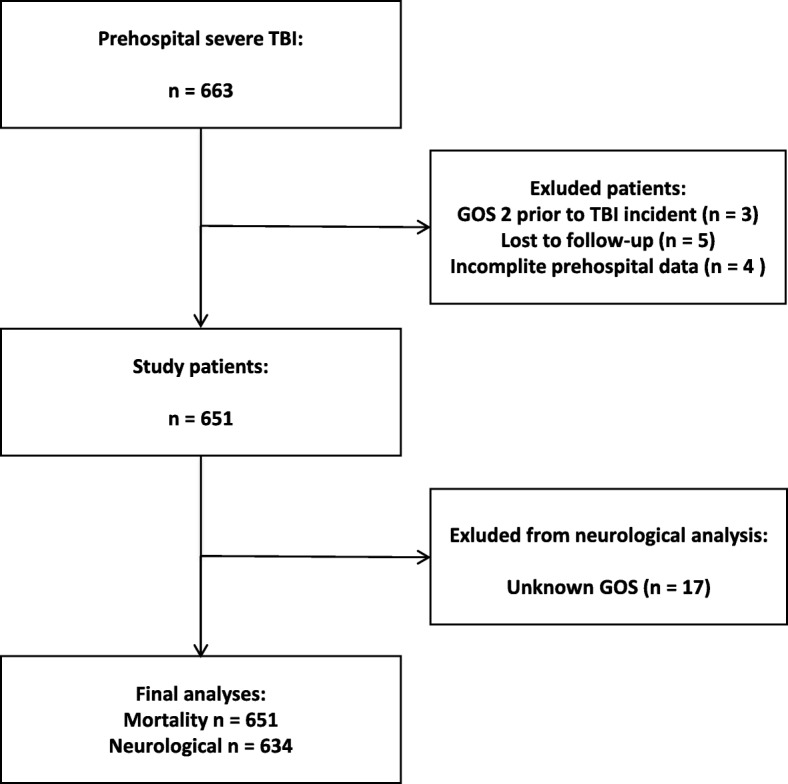
Flowchart

Six hundred and sixty-three patients met the inclusion criterion. The mortality data for 651 patients and neurological outcome data for 634 patients were available for analysis (Fig. 1). Information on the sociodemographic patient characteristics, mechanism of injury, response and total prehospital times is provided in Table 1.

**Table 1:** General characteristics

	Median / %	Q_1_-Q_3_
Age (y)	50	30–64
Male	74%	
Mechanism of injury		
Fall	38%	
Traffic accident	24%	
Fall from a height (> 2 m)	12%	
Violence	9%	
Other	7%	
Unknown	9%	
1st EMS Unit on scene (minutes)	8	5–12
Total mission time (minutes)	69	53–92
GCS on-scene (range)	5 (3–15)	
Hypoxia		
On-scene	16%	
ER	4%	
Hypotension		
On-scene	3%	
ER	4%	
Physician	72%	
Airway secured	74%	

*GCS* Glasgow Coma Score, *ER* Emergency Room

*Hypoxia* SpO_2_ of ≤90%, *Hypotension* systolic blood pressure (SBP) of ≤90

The median on-scene GCS was 5 (≤ 8 in 90%, 9–13 in 8% and 14–15 in 2% of the patients). Patients in the latter two groups deteriorated either on scene or during transportation and were consequently eligible for inclusion. Hypoxia was present on scene in 16% of the patients and hypotension was documented in 3% of them. The incidence of hypoxia (4%) and hypotension (4%) was similar on arrival at the ED. An anaesthetist was present on scene in 72% of the cases and advanced airway management was performed in 74% of the patients. The airway of 97% of the patients was secured in the prehospital setting when an on-scene anaesthetist was present and in 16% of the patients who were not treated by a physician.

Higher age, lower on-scene GCS and the unavailability of an on-scene anaesthetist were associated with higher mortality in univariate analysis. The same variables (age, GCS, an on-scene anaesthetist), together with hypotension, were found to be significant factors for mortality in multivariable analysis (Table 2).

**Table 2:** Mortality regression analyses

	Univariate			Multivariable		
	OR	95% CI	*p*-value	OR	95% CI	*p*-value
Age	1.06	1.05–1.07	< 0.001	1.06	1.05–1.07	< 0.001
GCS On-scene	0.91	0.85–0.96	0.002	0.85	0.79–0.92	< 0.001
Hypoxia						
Not present	1					
On-scene	1.31	0.84–2.03	0.230	0.93	0.55–1.59	0.792
Hypotension						
Not present	1					
On-scene	2.03	0.78–5.31	0.149	3.92	1.08–14.23	0.038
Physician						
Not present	2.03	1.44–2.88	< 0.001	1.89	1.20–2.94	0.005
On-scene	1					

*GCS* Glasgow Coma Score, *OR* Odds ratio, *CI* Confidence Interval

*Hypoxia* SpO_2_ of ≤90%, *Hypotension* systolic blood pressure (SBP) of ≤90 mmHg

Lower age, a higher on-scene GCS and the presence of an on-scene anaesthetist were linked to good neurological outcomes in univariate analysis. Following multivariable analysis, all of these factors were demonstrated to be significantly associated with good patient outcomes (age, GCS, an on-scene anaesthetist), while hypotension was associated with poor outcomes (Table 3).

**Table 3:** Good neurological outcome regression analyses

	Univariate			Multivariable		
	OR	95% CI	*p*-value	OR	95% CI	*p*-value
Age	0.95	0.94–0.96	< 0.001	0.95	0.94–0.96	< 0.001
GCS On-scene	1.15	1.08–1.22	< 0.001	1.21	1.20–1.30	< 0.001
Hypoxia						
Not present	1					
On-scene	0.66	0.41–1.05	0.079	1.05	0.60–1.83	0.863
Hypotension						
Not present	1					
On-scene	0.44	0.14–1.34	0.148	0.19	0.04–0.82	0.026
Physician						
Not present	0.51	0.35–0.74	< 0.001	0.57	0.36–0.92	0.020
On-scene	1					

*GCS* Glasgow Coma Score, *OR* Odds ratio, *CI* Confidence Interval

*Hypoxia* SpO_2_ of ≤90%, *Hypotension* systolic blood pressure (SBP) of ≤90 mmHg

## Discussion

In this retrospective observational study, prehospital on-scene anaesthetist treating severe TBI patients was associated with lower mortality and better neurological outcome.

The results supports our previous finding following an evaluation of mortality and neurological outcomes in TBI patients [18, 19]. However, there is lack of consensus on the impact of physician-staffed EMS on trauma patients in the literature and results from existing studies are inconclusive [16, 17, 24–27].

Early definitive airway control has become an established principle in the management and resuscitation of critically injured patients. This practise is considered to be the standard of care, particularly in patients with head trauma as hypoxemia and hypercapnia can worsen brain injury [28].

Prehospital treatment (i.e., ensuring a secured airway, preventing hypoxemia and enabling controlled ventilation) administered by an on-scene anaesthetist was associated with the observed lower mortality and improved neurological outcome in patients in the current study.

Virtually all patients with severe TBI who were treated by an on-scene anaesthetist had their airways secured in the prehospital setting. This concurs with the finding of a recent study by Gellerfors et al., in which it was shown that prehospital tracheal intubation was completed rapidly, with high success rates and a low incidence of complications when performed by experienced anaesthetists [29].

It has been suggested that the dispatch of physician-staffed EMS could increase on-scene time (OST). It is likely that different prehospital treatment strategies (i.e., “scoop and run” and “stay and play”) and interventions (i.e., airway management performed on scene) influence the OST and, depending on the injury profile, impact on patient outcomes. The literature is also inconclusive regarding the effect of prehospital timeframes on the outcomes of patients with severe TBI [17, 24, 30]. Unfortunately, reliable prehospital OST data were not available in our study.

Hypotension has been shown to have a negative impact on TBI outcomes in previous studies [10, 12]. It has been suggested that SBP values higher than 90 mmHg may benefit patients with isolated, severe TBI [31–35]. Hypotension, or the lack of it, was seen to have a significantly negative impact on survival (i.e., increased mortality) and a significantly positive impact on neurological outcomes, respectively, on multivariable analysis in the current study.

When considering other individual prognostic factors, age is an important predictor of outcome after brain trauma. The elderly (typically defined as age higher than 64–70 years) have higher mortality and worse functional outcomes compared to younger patients with the oldest patients having the poorest outcomes [36–39]. A GCS score of 3 at presentation is associated with very poor outcomes. Similarly, an increase in mortality and the worsening of neurological outcomes has been demonstrated in patients with a GCS of ≤8 [40–42]. A prehospital assessment of the GCS has been found to be an important and reliable indicator of the severity of TBI and should ideally be measured prior to the administration of sedative or paralytic agents [4]. The assessment should be repeatedly conducted to determine improvement or deterioration over time [4]. The results of the current study are comparable with these earlier findings.

### Strengths and limitations

Strengths of the current study were that this was a population-based study and that all primary EMS mission patients with severe TBI were treated and cared for in the study university hospitals. The included patients were recruited based on a confirmed diagnosis of severe TBI on discharge. Lastly, the mortality data were obtained from the national statistical authority, Statistics Finland, which publishes official causes of death statistics.

A major limitation of this study is that, due to the design, the improved patient outcome can only be associated with the treatment provided by prehospital physician. To obtain prehospital data and timeframes, the study only included patients from primary EMS missions. Also, neurosurgical and intensive care advances were made as well as a new HEMS unit was implemented to one of the EMS system during the study period, all which should be taken into consideration when interpreting the results. The prehospital data were not originally documented for the purpose of this study, could not be independently verified and thus could have been biased. Continuous data on patient vital signs for the entire prehospital phase were unavailable. Accordingly, transient hypoxia or hypotension during the prehospital period could not be excluded with absolute certainty. Similarly, an eye assessment (pupils) was not recorded for all of the patients. Thus, all of the prognostic variables used in previous studies were not available for analysis in this study. It is possible that the deaths that occurred in the late stages of the follow-up period were unrelated to the prehospital index event, i.e., secondary disease or injury was the cause. The outcome evaluation was based on an evaluation of the patient records by without the ability to perform a clinical examination or with the help of a questionnaire.

## Conclusion

Prehospital on-scene anaesthetist treating severe TBI patients is associated with lower mortality and better neurological outcome.

## Appendix

**Table 4:** Comparison of the patients between the study locations

	Helsinki and Uusimaa		Tampere		
	Median / %	Q_1_-Q_3_	Median / %	Q_1_-Q_3_	*p*-value
GCS On-scene	4	3–7	5	3–7	0.139
Hypoxia					
On-scene	14.1%		18.2%		0.171
ER	1.4%		7.8%		< 0.001
Hypotension					
On-scene	3.3%		4.2%		0.558
ER	1.9%		4.3%		0.088

*GCS* Glasgow Coma Score, *ER* Emergency Room

*Hypoxia* SpO_2_ of ≤90%, *Hypotension* systolic blood pressure (SBP) of ≤90

## Abbreviations

ED: Emergency department
EMS: Emergency medical services
GCS: Glasgow Coma Scale
GOS: Glasgow Outcome Score
HEMS: Helicopter emergency medical service
TBI: Traumatic brain injury
